# Hermansky-Pudlak Syndrome Type 6 and Renal Failure: A Rare Genetic Disease

**DOI:** 10.7759/cureus.47970

**Published:** 2023-10-30

**Authors:** Hanadi Alhozali

**Affiliations:** 1 Faculty of Medicine, Department of Medicine, Nephrology Unit, King Abdulaziz University Hospital, Jeddah, SAU

**Keywords:** end-stage renal disease (esrd), inherited bleeding diathesis, oculocutaneous albinism, renal failure, hermansky-pudlak syndrome

## Abstract

Hermansky-Pudlak syndrome (HPS) is a group of 10 autosomal recessive inherited diseases. Most patients exhibit albinism with nystagmus, visual acuity loss, and a platelet storage pool deficiency with bleeding diathesis. The severity and variety of other clinical features depend on the HPS subtype. We report a 24-year-old male with end-stage renal disease (ESRD) of unknown etiology and a history of oculocutaneous albinism and bleeding diathesis. Two of his siblings also had oculocutaneous albinism. The diagnostic workup for renal impairment was unremarkable. Further genetic testing revealed a homozygous novel nonsense mutation in the *HPS6 *gene. Additionally, a heterozygous variant of uncertain significance was identified in the *HPS5* gene. Renal failure is an uncommon clinical feature of HPS. To our knowledge, this is the first case that describes the association of HPS types 5 and 6 with renal failure.

## Introduction

Hermansky-Pudlak syndrome (HPS) is a rare autosomal recessive genetic disease that was first reported in the 1950s in two unrelated patients by two different physicians, Hermansky and Pudlak [1]. Both patients had hypopigmentation, pigmented reticular cells in the bone marrow, ocular manifestations, and prolonged bleeding time being the only laboratory abnormality. One of the patients also had renal insufficiency and was found to have a horseshoe kidney during an autopsy. The first case was reported in Prague, with subsequent cases reported in Puerto Rico [2].

There are 11 known subtypes with varying severities and body system involvement [2,3]. Pennamen et al. recently described HPS type 11 in 2020 [4]. The prevalence of HPS is estimated at 1-9 per 1,000,000. Patients with HPS commonly have a bleeding diathesis, oculocutaneous albinism, visual acuity loss, and nystagmus. Additionally, they may have granulomatous colitis and pulmonary fibrosis. These manifestations may be attributable to defects in melanosomes, platelet-dense granules, and lysosomal abnormalities. As shown in the Appendices, the severity and variety of clinical manifestations depend on the HPS subtype [5,6].

HPS-associated genes encode components of four expressed protein complexes: adaptor protein-3 (AP-3) and biogenesis of lysosome-related organelles complex-1 (BLOC-1) through BLOC-3. Patients with HPS subtypes 1, 2, and 4 have severe disease phenotypes with pulmonary fibrosis, which develops at a young age. HPS subtypes 3, 5, and 6 express a protein that depends on the BLOC-2 complex (biogenesis of lysosome-related organelles complex-2). Therefore, to an extent, these subtypes bear some clinical resemblance to the other subtypes. The literature revealed several reports of HPS type 6, and the largest cohort was reported in an Israeli Bedouin family, with at least 20 affected individuals [7], followed by four cases from the United States [8], and three cases from the Arabian Peninsula [9]. All reported cases had a severe mutation affecting the production of BLOC-2, but renal involvement was not part of their phenotype. This report describes the first case of HPS type 5 heterozygous variant and HPS type 6 in a patient with renal failure.

## Case presentation

**Figure 1:**
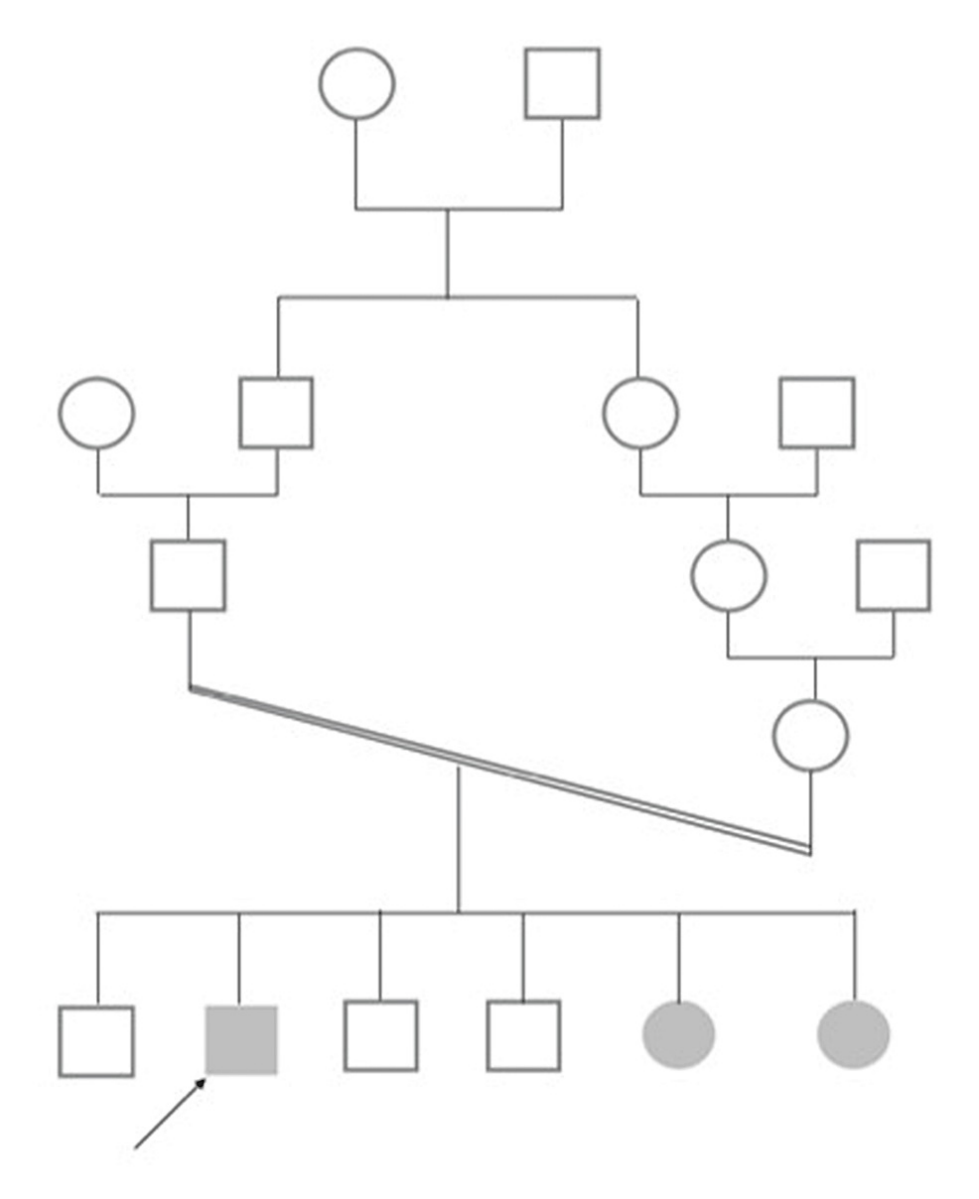
Patient’s pedigree family tree

A 24-year-old male with no relevant medical history presented with a three-month complaint of progressive abdominal pain, nausea, vomiting, and significant weight loss. Additionally, he had been complaining of shortness of breath on minimal exertion. Of note, he had a history of prolonged bleeding after tooth extractions and easy bruising. He was a known case of oculocutaneous albinism with two of his siblings reported to have the same condition and the arrow is the patient in his family tree (Figure 1). His family history was unremarkable for any chronic illness. On examination, his blood pressure was high with readings ranging from 165/100 to 183/103 mmHg (normal reading: less than 130/80); however, the rest of the physical examination was unremarkable. Further, he was noted to have a dark olive skin color but a significantly lighter complexion compared to the rest of his family, which can be explained by the mixture of his ethnicity and albinism. He also had light brown hair and horizontal nystagmus.

Laboratory investigations revealed evidence of advanced renal impairment, and an ultrasound examination of the abdomen showed small echogenic kidneys. Other basic investigations such as liver function and other endocrine functions were unremarkable. All secondary workup, such as autoimmune, viral serology, antineutrophil cytoplasmic antibodies (ANCA), and paraproteinemia screening, performed to determine the etiology of the renal failure revealed negative results. A kidney biopsy was not performed due to a low diagnostic yield and a high risk of bleeding in a state of uremia. Renal replacement therapy in the form of hemodialysis was initiated, and five months later, he underwent a living-related kidney transplant from his brother. The post-transplant period was uneventful.

Investigation

HPS was suspected based on the constellation of classic disease symptoms and signs. A pulmonary function test and computed tomography (CT) of the thorax ruled out pulmonary fibrosis. Genetic testing was performed as part of the diagnostic workup, and the patient consented to molecular testing. A blood sample was shipped to CENTOGENE Laboratory in Germany for genetic testing. His sisters (aged 12 and 18 years) were assessed, and a clinical diagnosis of oculocutaneous albinism was made. However, his mother refused to proceed with genetic testing on both siblings. Blood and urine samples were obtained to screen for renal disease, and the results were normal for both siblings.

Sequence analysis

Genomic DNA was extracted from peripheral blood leukocytes, and the patient was screened for mutations in *HPS1*, *AP3B1*, *HPS3*, *HPS4*, *HPS5*, *HPS6*, *DTNBP1*, and *BLOC1S3*. Sequencing of complementary DNA was performed using next-generation sequencing (NGS) technology, followed by Sanger sequencing. The entire coding regions were sequenced, including intronic flanking regions. Sequence data were compared and aligned with the Hg 19 human reference genome. Variants were also assessed for pathogenicity using validated in-house software.

Genetic testing

Mutation Analysis

**Figure 2:**
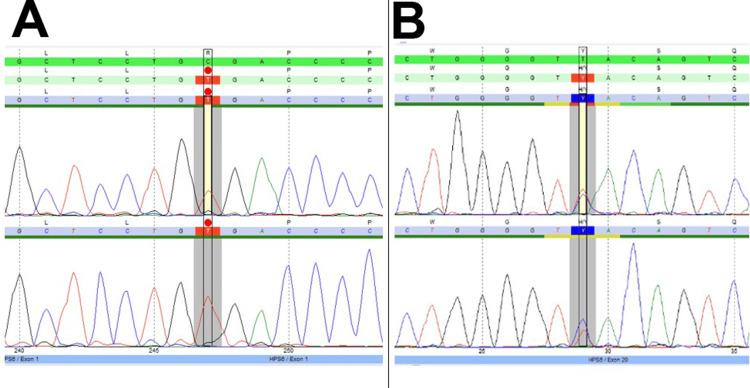
Molecular findings: sequencing chromatogram A: homozygous c.139C>, B: heterozygous c.2866T>C

We identified a homozygous novel nonsense mutation in the *HPS6* gene, creating a premature stop codon and interrupting the reading frame c.139C>T (p.Arg47X) (Figure 2a). This mutation affected the only exon of the *HPS6* gene, resulting in a truncated protein. In addition, another heterozygous variant of uncertain significance was identified in the *HPS5* gene c.2866T>C (p.Tyr956His) (Figure 2b). The changes in the *HPS5* gene were in a moderately conserved nucleotide and a highly conserved amino acid position, with moderate physicochemical differences between the exchanged amino acids (Alamut v.2.7.1); however, no functional or expression analyses were performed to prove its pathogenicity.

Detailed genetic test report

We detected a homozygous variant in the *HPS6* gene, c.139C>T (p.Arg47*). The reading frame is interrupted by a premature stop codon. This variant is reported in the Exome Aggregation Consortium (ExAC) with a frequency of 0.000027, i.e., in one among 36,744 alleles (ExAC database). This is the first time we have detected this variant, and it is so far not listed in CentoMD®. It is classified as likely pathogenic (class 2) according to the recommendations of CENTOGENE and American College of Medical Genetics (ACMG). We also detected a heterozygous variant in the *HPS5* gene, c.2866T>C (p.Tyr956His). It is located in a moderately conserved nucleotide and highly conserved amino acid position, with moderate physicochemical differences between the exchanged amino acids (Alamut v.2.7.1). Software analyses show inconsistent predictions: PolyPhen-2 and MutationTaster indicate that this variant is probably damaging, whereas Align-GVGD and SIFT predict toleration. This variant is reported in the Exome Aggregation Consortium with a frequency of 0.0007, i.e., in 87 among 120,766 alleles (ExAC database). The Exome Sequencing Project describes it with a frequency of 0.001 in the European American population (ESP - Alamut v.2.7.1). It is also described in the 1,000 Genomes Browser with an average allele frequency of 0.0002, rising up to 0.0049 in Sri Lankan Tamil from the UK population. This is the first time we have detected this variant, and it is so far not listed in CentoMD®. It is classified as a variant of uncertain significance (class 3) according to the recommendations of CENTOGENE and ACMG. Homozygous or compound heterozygous pathogenic variants in the *HPS6* gene cause Hermansky-Pudlak syndrome type 6 (HPS6, Online Mendelian Inheritance in Man (OMIM) 614075). HPS6 presents with features of HPS, including ocular or oculocutaneous albinism, reduced visual acuity, horizontal nystagmus, easy bruising of soft tissues, epistaxis, and prolonged bleeding. Pulmonary fibrosis has not been found to develop in HPS6. The course of HPS with no pulmonary involvement is mild (ORPHA231512). Based on the obtained result, a genetic diagnosis of HPS6 is very likely.

## Discussion

In this report, we describe a case of partial oculocutaneous albinism in a patient with renal failure in whom genetic testing confirmed HPS type 6. Other clinical features of HPS, such as granulomatous colitis and pulmonary fibrosis, were not present. Hermansky-Pudlak syndrome is a heterogeneous genetic disorder with 10 genes identified to date, and it is responsible for 10 different phenotypes [10]. The identification of these disease subtypes guides management and predicts prognosis, as patients with the HPS2 and HPS4 subtypes tend to have severe phenotypes compared to those with HPS3, HPS5, and HPS6, who typically present milder phenotypes. A genotype-phenotype correlation is not evident among different HPS subtypes. Genes for HPS6, HPS3, and HPS5 encode for “biogenesis of lysosome-related organelles complex-2 (BLOC-2),” suggesting a common pathogenic mechanism and explaining their phenotypic similarities [11,12]. These lysosome-related organelle complexes are critical for intracellular protein trafficking with subsequent accumulation of a ceroid-like material known to infiltrate tissues (i.e., lungs, colon, and kidney) in HPS, but the underlying cause of renal impairment remains unknown [13].

In our patient’s case, the mechanism underlying the etiology of renal disease could not be determined by clinical and paraclinical investigations. The patient’s genetic testing was done in 2016, and a review of the medical literature at that time did not identify any possible pathological link between HPS and renal disease. However, Schenk et al. presented a valid zebrafish model that highlights the previously underestimated relevance of renal disease in HPS [13]. Moreover, HPS proteins were found to have a high renal expression with active transcription of HPS1, HPS3, HPS4, and HPS5 in human podocyte cell culture [14]. Renal histological diagnosis would have been helpful to rule out other possible pathogenesis, but a biopsy was not performed for reasons mentioned previously.

Gordillo et al. reported a child with chronic kidney disease and HPS, with subsequent kidney biopsy showing focal segmental glomerulosclerosis not otherwise specified [15]. Additionally, a histopathology examination showed chronic diffuse tubulopathy (tubular cytoplasmic droplets) and acute tubular injury. Other investigators reported IgA nephropathy with ANCA-positive glomerulonephritis in patients with HPS [15,16]. In all, renal manifestations in HPS might be secondary to etiologies that are totally unrelated to the pathogenies of the condition.

Different types of mutation have been reported in the *HPS6* gene, with the majority being nonsense and frameshift [8]. Our patient had a diagnosis of HPS type 6 with novel homozygous pathogenic gene variants, resulting in a truncated protein in addition to heterozygous changes in the *HPS5* gene of unknown clinical significance. This patient’s case is unique in that he had mutations in the *HPS6* gene and a heterozygous variant in the *HPS5* gene, which both encode for the same protein. The presence of both mutations may explain the severe clinical phenotype, with our patient presenting with renal failure. Nevertheless, further investigational reports are warranted to determine any relationship between HPS and renal failure. We believe that in order to detect renal impairment, it is critical to perform a family annual screening that includes an analysis of blood and urine samples in the patient’s siblings with oculocutaneous albinism.

## Conclusions

Herein, we report a 24-year-old male patient with oculocutaneous albinism and easy bruising who presented with evidence of ESRD. Clinical suspicion based on the presence of albinism, a bleeding disorder, and renal disease prompted a genetic investigation. Genetic testing identified a homozygous novel nonsense mutation in the *HPS6* gene. Additionally, another heterozygous variant of uncertain significance was identified in the *HPS5* gene.

The *HPS5* and *HPS6* genes encode the same protein, which may contribute to the severe phenotype characterized by renal impairment. Renal histological evaluation may prove useful in determining the etiology of renal failure. However, in our case, a biopsy was not performed due to a low diagnostic yield in the presence of small fibrotic kidneys and an increased risk of uremic bleeding post-procedure. Genetic testing is increasingly used to inform clinical management of kidney disease of unknown etiology and has led to improved diagnostics, disease surveillance, choice of therapy, and family counseling. Further research is required to confirm the exact pathogenic link between renal failure and HPS types 5 and 6.

## Appendices

Table 1 summarizes the clinical features of different types of Hermansky-Pudlak syndrome.

**Table 1:** Summary of the clinical features of different types of Hermansky-Pudlak syndrome a: decreased lung compliance, b: interstitial lung disease HPS: Hermansky-Pudlak syndrome, OMIM: Online Mendelian Inheritance in Man

System involved/type	HPS1 OMIM 604982	HPS2 OMIM 608233	HPS3 OMIM 614072	HPS4 OMIM 614073	HPS5 OMIM 6140 74	HPS6 OMIM 614075	HPS7 OMIM 614076	HPS8 OMIM 614077	HPS9 OMIM 614171	HPS10 OMIM 617050
Ocular hypopigmentation		+	+	+	+	+	+	+	+	+
Facial dysmorphism		+								+
Cardiomyopathy	+									
Pulmonary fibrosis	+	+					+^a^		+	+^b^
Granulomatous colitis	+						+			
Renal failure	+									
Albinism	+	+	+	+	+	+	+	+	+	+
Bleeding diathesis	+	+	+	+	+	+	+	+		+
Seizures/neurodevelopmental delay		+							+	+
Immune deficiency		+							+	+
Skeletal dysplasia										+
